# A prospective study of the importance of enteric fever as a cause of non-malarial febrile illness in patients admitted to Chittagong Medical College Hospital, Bangladesh

**DOI:** 10.1186/s12879-016-1886-3

**Published:** 2016-10-13

**Authors:** Rapeephan R. Maude, Aniruddha Ghose, Rasheda Samad, Hanna K. de Jong, Masako Fukushima, Lalith Wijedoru, Mahtab Uddin Hassan, Md Amir Hossain, Md Rezaul Karim, Abdullah Abu Sayeed, Stannie van den Ende, Sujat Pal, A. S. M. Zahed, Wahid Rahman, Rifat Karnain, Rezina Islam, Dung Thi Ngoc Tran, Tuyen Thanh Ha, Anh Hong Pham, James I. Campbell, H. Rogier van Doorn, Richard J. Maude, Tom van der Poll, W. Joost Wiersinga, Nicholas P. J. Day, Stephen Baker, Arjen M. Dondorp, Christopher M. Parry, Md Abul Faiz

**Affiliations:** 1 Mahidol-Oxford Tropical Medicine Research Unit (MORU), Faculty of Tropical Medicine, Mahidol University, Bangkok, Thailand; 2 Chittagong Medical College Hospital, Chittagong, Bangladesh; 3 Department of Internal Medicine, Division of Infectious Diseases and Center for Infection and Immunity Amsterdam (CINIMA), and Center for Experimental Molecular Medicine (CEMM), Academic Medical Center, University of Amsterdam, Amsterdam, The Netherlands; 4 Clinical Sciences, Liverpool School of Tropical Medicine, Liverpool, UK; 5 Wellcome Trust Major Overseas Programme, Oxford University Clinical Research Unit (OUCRU), Hospital for Tropical Diseases, Ho Chi Minh City, Vietnam; 6 Nuffield Department of Medicine, Centre for Tropical Medicine and Global Health, Oxford University, Oxford, UK; 7 Clinical Research Department, London School of Hygiene and Tropical Medicine, Keppel Street, London, WC1E 7HT UK; 8 School of Tropical Medicine and Global Health, Nagasaki University, Nagasaki, Japan; 9 Malaria Research Group and Dev Care Foundation, Chittagong, Bangladesh

**Keywords:** Non-malaria febrile illness, Bangladesh, Enteric fever

## Abstract

**Background:**

Fever is a common cause of hospital admission in Bangladesh but causative agents, other than malaria, are not routinely investigated. Enteric fever is thought to be common.

**Methods:**

Adults and children admitted to Chittagong Medical College Hospital with a temperature of ≥38.0 °C were investigated using a blood smear for malaria, a blood culture, real-time PCR to detect *Salmonella* Typhi, *S.* Paratyphi A and other pathogens in blood and CSF and an NS1 antigen dengue ELISA.

**Results:**

We enrolled 300 febrile patients with a negative malaria smear between January and June 2012: 156 children (aged ≤15 years) and 144 adults with a median (interquartile range) age of 13 (5–31) years and median (IQR) illness duration before admission of five (2–8) days. Clinical enteric fever was diagnosed in 52 patients (17.3 %), lower respiratory tract infection in 48 (16.0 %), non-specific febrile illness in 48 (16.0 %), a CNS infection in 37 patients (12.3 %), urinary sepsis in 23 patients (7.7 %), an upper respiratory tract infection in 21 patients (7.0 %), and diarrhea or dysentery in 21 patients (7.0 %). Malaria was still suspected in seven patients despite a negative microscopy test. *S.* Typhi was detected in blood by culture or PCR in 34 (11.3 %) of patients. Of note *Rickettsia typhi* and *Orientia tsutsugamushi* were detected by PCR in two and one patient respectively. Twenty-nine (9 %) patients died during their hospital admission (15/160 (9.4 %) of children and 14/144 (9.7 %) adults). Two of 52 (3.8 %) patients with enteric fever, 5/48 (10.4 %) patients with lower respiratory tract infections, and 12/37 (32.4 %) patients with CNS infection died.

**Conclusion:**

Enteric fever was confirmed in 11.3 % of patients admitted to this hospital in Bangladesh with non-malaria fever. Lower respiratory tract and CNS infections were also common. CNS infections in this location merit more detailed study due to the high mortality.

## Background

Febrile illnesses are a common cause of admission to hospitals in resource limited settings but the limited availability of routine diagnostic microbiology testing means that the causes are often incompletely understood. Intensive diagnostic studies performed over short periods have proved to be a useful approach to determine the relative importance and resistance patterns of common pathogens and the identification of new infections [1–4]. A greater understanding the clinical epidemiology of local infections enables public health authorities to focus limited resources.

In Chittagong Division, Bangladesh, malaria is an important reason for people with fever to seek medical advice and hospital admission and is generally diagnosed using a malaria blood smear and/or a rapid diagnostic test [5]. For patients in whom malaria has been excluded, enteric (typhoid) fever is believed to be common and dominated by antimicrobial resistant organisms [6–13]. The importance of other diseases, such as melioidosis, rickettsiosis and leptospirosis, are not well defined [14]. There is serological evidence of exposure to *Burkholderia pseudomallei* (causative agent of melioidosis), *Orientia tsutsugumushi* and *Rickettisa typhi* (the causative agent of rickettsiosis) but very few reports of confirmed illness [15, 16].

We conducted a prospective study to assess the etiology of non-malarial febrile illness in adults and children admitted to Chittagong Medical College Hospital, a 1000-bed regional teaching hospital. Our aim was to determine the proportion of patients with enteric fever. We also sought to determine the range of other clinical syndromes, causative agents and to document patient outcomes. An evaluation of typhoid rapid diagnostic tests in the patients in this study has been reported previously [17].

## Methods

### Study site and population

Chittagong Medical College Hospital (CMCH) is a 1000-bed teaching hospital in Chittagong Division of Bangladesh. It provides inpatient and outpatient medical, surgical, pediatric and gynecology services. Each year there are approximately 700,000 outpatient visits, 700,000 emergency department visits and 600,000 admissions (500,000 adults and 100,000 children). Bed occupancy for medical wards runs at more than 100 % with extra beds provided on a daily basis.

Chittagong is the second largest city of Bangladesh. It is located in southeastern Bangladesh with a population in the metropolitan area of over 6.5 million people. Chittagong has a tropical monsoon climate with average temperatures ranging between 21.6 and 30.2 °C and average humidity of 78 %. The climate varies from tropical monsoon climate from March to November and cool and dry winters from December to February [18].

### Patients and clinical methods

Patients admitted to CMCH between 15^th^ January 2012 and 5^th^ July 2012 were considered for enrollment. Eligibility criteria were age more than 6 months, documented axillary temperature ≥38 °C, a reported history of fever less than 2 weeks, a negative malaria smear and written informed consent given by patient or by the parents or caregiver if a child (age <16 years). Each study day all the patients admitted to the “on-take” wards were reviewed and those with a history of fever of the appropriate duration and a negative malaria smear had their temperature measured on at least one occasion. Eligible patients were then asked if they consented to participate in the study. Demographic and clinical information was recorded on a case record form at the time of admission and during the course of hospitalization.

A final diagnosis was made by the study team based on clinical presentation, basic laboratory results and microbiology results. A diagnosis of probable enteric fever was considered if the patient presented with a febrile illness of >3 days duration and two or more of the following clinical symptoms: the presence of abdominal symptoms (abdominal pain, diarrhea, constipation, nausea or vomiting), a documented fever of ≥39 °C; hepatomegaly and/or splenomegaly; a low or normal white cell count; elevation of liver enzymes (aspartate transaminase, alanine transaminase) three times above the normal range; combined with a slow defervescence with ceftriaxone treatment (the standard antibiotic used for hospital admitted febrile patients with suspected enteric fever) or a clear typhoid complication and no alternative confirmed diagnosis was established.

### Laboratory methods

Blood was taken for complete blood count, urea and creatinine, aspartate transaminase (AST), alanine transaminase (ALT), malaria smear, a single BactAlert® blood culture, and a sample for real time PCR assays and serology. Cerebrospinal fluid was taken at the discretion of the responsible physician in suspected central nervous system infection as part of routine practice. Usually only small volumes of CSF were taken, sufficient for a cell count and biochemistry but insufficient for any further investigations. EDTA whole blood, serum, and any residual CSF (where there was sufficient) were stored at −20 °C for later analysis by PCR and serology at the Oxford University Clinical Research Unit (OUCRU), Ho Chi Minh City, Vietnam.

### Bacterial culture

Blood was taken for culture within 24 h of admission, when possible before antimicrobial therapy was started in hospital. A volume of blood, 5–12 mL in adults and 1–12 mL for children, was inoculated into an adult or pediatric BactAlert® blood culture bottle. The exact volume of blood added was determined by weighing the bottle before and after blood inoculation. They were incubated aerobically in a BactAlert® automated system (bioMérieux, Marcy l’Etoile, France) at 37 °C for 5 days. A Gram stained smear was prepared from the broth of bottles that were positive and which was also sub-cultured onto 5 % sheep blood agar and chocolate agar (Oxoid, Basingstoke, UK) incubated in a candle jar and MacConkey agar (Oxoid, Basingstoke, UK) incubated in air for 48 h. Bacterial isolates were identified by standard methods including biochemical test using API test strips (bioMérieux, Marcy l’Etoile, France) and agglutination with specific antisera (Biorad, Hemel Hempstead, UK) [19].

Antimicrobial susceptibility tests were determined using disc diffusion with results interpreted according to Clinical Laboratory Standards Institute (CLSI) guidelines [20]. Isolates of *Salmonella* were tested with chloramphenicol (30 μg), ampicillin (10 μg), trimethoprim-sulphamethoxazole (1.25/23.75 μg), ceftriaxone (30 μg), ciprofloxacin (5 μg), and nalidixic acid (30 μg). The minimum inhibitory concentration (MIC) was determined by E- test according to the manufacturer’s guidelines (AB Biodisk, Solna, Sweden) against ciprofloxacin, ceftriaxone and azithromycin. *Salmonella* breakpoints for ciprofloxacin were: susceptible ≤0.06 μg/mL; intermediate >0.06–≤0.5 μg/mL; and resistant ≥1.0 μg/mL. A cut-off for susceptibility of ≤16 μg/mL was used for azithromycin. Other Enterobacteriacae were additionally tested against ceftazidime (30 μg), imipenem (10 μg) and gentamicin (10 μg). Isolates non-susceptible to ceftriaxone or ceftazidime were tested for extended spectrum beta-lactamase (ESBL) activity by comparing inhibition zone sizes of cefpodoxime, ceftriaxone and ceftazidime with and without clavulanic acid with a difference of 5 mm or more indicating ESBL activity. *Streptococcus pneumoniae* was tested with oxacillin (1 μg). and *Staphylococcus aureus* was tested with penicillin (10U) and cefoxitin (30 μg) (resistance indicating meticillin resistance). *Escherichia coli* ATCC25922 and *Staphylococcus aureus* ATCC25923 were used as control strains for these assays. All media and tests were subject to regular internal quality assessment. Bacterial isolates were stored on beads in glycerol at −80 °C and later transferred to the OUCRU, Vietnam for re-confirmation of their identification and susceptibility results.

### Serology

The admission serum was analysed by ELISA for dengue NS1 antigen using a PanBio Kit (PanBio, Sinnamon Park, Australia) according to the manufacturer’s instructions.

### Nucleic acid amplification tests

DNA was extracted from the stored whole blood samples (2 mL in adults and 1 mL from children) with a QIAmp DNA mini kit (Qiagen, Manchester, UK). A real-time PCR for *S.enterica* Typhi and *S.enterica* Paratyphi was performed using 25 μL reactions containing 5 μL of extracted DNA targeting *STY0201* (Putative fimbrial adhesion in *S.* Typhi CT18) or *SSPA2308* (hypothetical protein in SPA AKU-12601) as previously described [21]. Probe-based real-time PCR was also performed on the DNA extracted from blood to detect *Leptospira* spp, *R.typhi* and *O.tsustugamushi* [22–24]. Low-positive plasmid controls determined adequate detection limits of each assay.

Total nucleic acid was isolated from 100 μL of CSF specimens using the automated easyMAG® system (bioMérieux), and diagnostic PCRs were performed [25]. Four real-time PCR protocols were used for detection of *S pneumoniae*, *H influenzae* type B, *Neisseria meningitidis*, and *Streptococcus suis*. Real time-PCRs were used to detect herpes simplex virus 1 and 2, varicella zoster virus, enteroviruses (generic and 71-specific) [26], and human parechoviruses (generic).

### Analysis

This was an observational study intended to serve as the basis for future studies with larger sample sizes and/or interventions. The true incidence of patients presenting with enteric fever in the CMCH is unknown. With a sample of at least 250 patients we would be able to estimate a disease prevalence of 5 % with a confidence interval (CI) of ± 2.8 %.

Demographic and clinical features were described for the whole cohort and within the stratified age categories of <5 years, 5–15 years and ≥16 years. Continuous data were described using median and inter-quartile range and compared using the Mann Whitney *U*-test. Proportions were compared with the Chi-squared test or the Fisher’s exact test as appropriate. Age, sex, duration of illness prior to admission to hospital, and the clinical syndrome were evaluated for association with in-hospital death through a univariate analysis. A multivariate logistic regression model controlling simultaneously for the effects of confounding included variables associated with the outcome of death (*p* < 0.10) as well as a priori factors age, sex and duration of illness prior to admission. Analysis was performed using SPSS version 21 (SPSS inc, Chicago, USA). The dataset supporting the conclusions of this article is available from the corresponding author.

## Results

### Demographics and clinical features

We enrolled 304 eligible febrile patients in this study. It was not possible to take blood from one patient and in three patients, who were blood culture negative, there was insufficient blood for PCR amplification. These four patients were excluded from this analysis. Of the 300 patients analyzed 156 were children (age ≤15 years) and 144 were adults. The median (interquartile range, range) age was 13.5 (5.0–31.0, 0.5–89) years and the median duration of illness before admission was five (IQR, 2–8; range, 1–14) days. A history of prior antimicrobial therapy was reported in 185 (61.7 %) of patients including 96 (61.5 %) of children and 89 (61.8 %) of adults.

A final clinical suspected or microbiologically confirmed diagnosis of enteric fever was made in 52 (17.3 %; 95 % Confidence Interval 13.5–22.0 %) patients. The other common clinical syndromes diagnosed were: lower respiratory tract infection in 48 patients (16.0 %), non-specific febrile illness in 48 patients (16.0 %), a CNS infection in 37 patients (12.3 %), urinary sepsis in 23 patients (7.7 %), upper respiratory tract infection in 21 patients (7.0 %), and diarrhea (including dysentery) in 21 patients (7.0 %). Malaria was clinically suspected in seven patients, despite a negative malaria smear, although all had received prior anti-malarial treatment. Two subsequently had a malaria positive rapid diagnostic test result. There were a 14 patients who fulfilled the clinical criteria for enteric fever but had a very rapid response to ceftriaxone treatment that was not typical for enteric fever. They were classified as another syndrome, most commonly as a non-specific febrile illness. The demographic features and clinical syndromes diagnosed in the patients according to age ranges are shown in Table 1.

**Table 1:** Demographic features and clinical syndromes of 300 patients admitted to CMCH with fever

Variable^a^	All	<5 years	5–15 years	>15 years
Number	300	70	86	144
Duration of illness before admission (median (IQR) days)	5 (2–8)	2 (1–5)	5 (3–10)	5 (3–8)
Male	173 (57.7)	37 (52.9)	56 (65.1)	80 (55.6)
Clinical syndrome:				
Enteric fever	52 (17.3)	0 (0)	19 (22.1)	33 (22.9)
Lower respiratory tract infection	48 (16.0)	13 (18.6)	5 (5.8)	30 (20.8)
Non-specific febrile illness	48 (16.0)	10 (14.3)	20 (23.3)	18 (12.5)
Central nervous system infection	37 (12.3)	12 (17.1)	13 (15.1)	12 (8.3)
Urinary tract infection	23 (7.7)	2 (2.9)	3 (3.5)	18 (12.5)
Upper respiratory tract infection	21 (7.0)	13 (18.6)	7 (8.1)	1 (0.7)
Diarrhea or dysentery	21 (7.0)	16 (22.9)	4 (4.7)	1 (0.7)
Malaria	7 (2.3)	0 (0)	3 (3.5)	4 (2.8)
Hepatobiliary	7 (2.3)	0 (0)	4 (4.7)	3 (2.1)
Skin and soft tissue infection	4 (1.3)	2 (2.9)	0 (0)	2 (1.4)
Dengue	3 (1.0)	0 (0)	1 (1.2)	2 (1.4)
Sepsis syndrome	3 (1.0)	0 (0)	0 (0)	3 (2.1)
Septic arthritis	2 (0.7)	0 (0)	1 (1.2)	1 (0.7)
Nephrotic syndrome	2 (0.7)	0 (0)	2 (2.4)	0 (0)
Dental abscess	1 (0.3)	0 (0)	0 (0)	1 (0.7)
Other^b^	17 (5.7)	2 (2.9)	4 (4.7)	12 (8.3)
Duration of admission (median (IQR) days)	4 (2–8)	4 (2–19)	7 (4–27)	4 (2–5)
Mortality (Number (%))	29 (9.7)	8 (11.4)	7 (8.1)	14 (9.7)

^a^Results are number (%) or median (Inter-quartile range)

^b^Haematalogical malignancy (6); autoimmune disease (4); post-streptococcal glomerulonephritis (2); cerebrovascular accident (2); post myocardial infarction (1); suspected brain tumor (1); costochondritis (1)

### Microbiological diagnoses

The median (IQR) volume of blood cultured was 2.2 (1.7–2.9) mL in children <5 years; 5.2 (4.2–5.8) mL in children 5–9 years; 6.6 (5.5–9.9) in children 10–15 years; and 9.2 (8.4–10.3) in those ≥16 years. A microbiological diagnosis was confirmed in 57 (19 %) patients as outlined in Table 2. All of the positive blood cultures grew just one organism. *Salmonella* Typhi was most prevalent organism detected in this study and accounted for 34 cases (11.3 %; 95%CI 8.2–15.5 %). Nineteen (6.3 %) patients had a positive blood culture for *S.* Typhi, including three patients also PCR amplification positive for *S.*Typhi in blood. Fifteen (5.0 %) patients with a negative blood culture were PCR amplification positive for *S.* Typhi in blood. The diagnosis was therefore confirmed in 34/52 (65.4 %) of patients in whom the diagnosis of enteric fever was suspected. No patient was positive for *Salmonella* Paratyphi A by blood culture or by PCR. A Gram-negative bacillus was found in the blood culture of a patient clinically diagnosed as enteric fever and a Gram positive diplococcus was observed in the blood culture of an adult with pneumonia but neither grew on sub-culture. Two adults were PCR amplification positive for *Rickettsia typhi* and one was PCR amplification positive for *Orientia tsutsugamushi*. All patients were PCR amplification negative for *Leptospirosis*. A further two adults were positive for dengue NS1 antigen in the admission serum sample. In the 37 patients with a clinically diagnosed CNS infection there was sufficient CSF sample available for further examination in 12. These were positive in four children: by PCR for *Neiserria meningitidis* in two, *Streptococcus pneumoniae* in one and one was IgM positive for Japanese encephalitis virus.

**Table 2:** Pathogens detected in 300 adults and children admitted to CMCH with fever

Pathogens	All	<5 years	5–15 years	>15 years
*Staphylococcus aureus*	2	1	1	0
*Streptococcus pneumoniae*	2	1	1	0
*Streptococcus acidominimus*	1	0	0	1
*Enterococcus spp.*	1	0	0	1
*Escherichia coli*	2	0	0	2
*Enterobacter cloacae*	2	0	0	2
*Klebsiella pneumoniae*	1	0	0	1
*Salmonella enterica* serotype Typhi				
Blood culture	19	0	8	11
PCR	18^a^	0	1	17
*Burkholderia cepacia*	3	3	0	0
*Acinetobacter* spp.	1	0	1	0
*Neisseria meningitdis*	2	0	2	0
*Rickettsia typhi*	2	0	0	2
*Orientia tsutsugamushi*	1	0	0	1
Dengue	2	0	0	2
Japanese encephalitis virus	1	1	0	0
Total	57/300 (19)	6/70 (9)	13/86 (15)	38/144 (26)

^a^Three also positive by blood culture

### Antimicrobial susceptibility results

Antimicrobial susceptibility results were available for 18 of the *S.* Typhi isolates. All 18 had intermediate susceptibility to ciprofloxacin, six were additionally multidrug-resistant (MDR) exhibiting resistance to chloramphenicol, ampicillin and co-trimoxazole but all were susceptible to ceftriaxone and azithromycin. Among the other Enterobacteriacae, one *E.coli*, one *K.pneumoniae* and one *E.cloacae* were resistant to ceftriaxone and Extended Spectrum Beta Lactamase (ESBL) positive. The *E.coli* and *K.pneumoniae* were additionally resistant to ciprofloxacin and the *K.pneumoniae* to gentamicin but all were susceptible to imipenem. The *S.pneumoniae* was penicillin susceptible and one of the two *S. aureus* was meticillin resistant.

### Outcome

A variety of empirical antimicrobials were employed for treatment. Most patients diagnosed with enteric fever were initially treated with ceftriaxone with a step down to oral azithromycin. The median duration of hospital stay was four (IQR 2–7, range 0–29) days.

A total of 29 (9.7 %) patients died during their hospital admission: 15/156 (9.6 %) of the children and 14/144 (9.7 %) adults. The mortality was 2/52 (3.8 %) patients with enteric fever; 5/48 (10.4 %) patients with lower respiratory tract infection; and 12/37 (32.4 %) patients with encephalitis/meningitis. There was no association with a history of antimicrobial use before hospital admission and mortality (*p* > 0.5). The association of age, sex, duration of illness before admission and the diagnosis of the four commonest clinical syndromes with fatal outcome are shown in Table 3. In a multivariate analysis including all of these variables a diagnosis of a CNS infection was independently associated with a fatal outcome (OR 6.81 (95 % CI 2.77–16.76; *p* < 0.001). Of the 12 patients with a CNS infection who died, sufficient CSF was available for pathogen detection in only two of which one was positive for *S. pneumoniae*.

**Table 3:** Univariate analysis of factors associated with a fatal outcome

Covariate	Died	Survived	*p*
	*n* = 29	*n* = 271	
Age (years)^a^	13 (4–45)	13 (5–30)	0.699
Male^b^	15 (51.7)	158 (58.3)	0.193
Days ill prior to admission^a^	5 (2–8)	4 (3–6)	0.713
Enteric fever^b^	2 (6.9)	50 (18.5)	0.193
Lower respiratory tract infection^b^	5 (17.2)	43 (15.9)	0.791
Non-specific febrile illness^b^	0 (0)	48 (17.7)	0.007
Central nervous system infection^b^	12 (41.4)	25 (9.2)	<0.001

^a^Median (Inter-quartile range)

^b^Number (%)

## Discussion

In this study of febrile adults and children admitted to hospital in Chittagong enteric fever was identified as the most common clinical syndrome responsible for almost one in five admissions. The diagnosis was confirmed by isolation of *S.* Typhi in blood culture and/or by real time PCR amplification from blood for *S.* Typhi in 11 % of admissions and two thirds of suspected cases. Making a confident clinical diagnosis of enteric fever in the absence of laboratory confirmation is difficult. We used a clinical algorithm to define patients with probable enteric fever but excluded a number of patients with a suggestive picture who did not respond to ceftriaxone in the slow manner in which patients with enteric fever often do. We also assumed that the detection of *S.* Typhi DNA in the peripheral blood by the real-time PCR assay indicates active disease although it is possible that the DNA may persist in the blood from a past infection. In the absence of an alternative suitable reference standard it is difficult to be completely confident that these approaches are correct.

Typhoid fever was most commonly diagnosed in school aged children and young adults in this group of hospitalized patients but was not diagnosed in children aged <5 years. This is in contrast to community-based studies in other sites in Bangladesh where typhoid was found in children <5 years [6–8]. In these community-based studies with active surveillance by blood cultures of children <5 years in clinics, most cases with blood culture confirmed typhoid fever did not require admission to hospital. This may suggest that more severe typhoid disease which requires hospital admission occurs in older age groups. Of the 18 tested *S.* Typhi isolates all demonstrated intermediate susceptibility to ciprofloxacin and one third were multidrug resistant. This is consistent with other studies originating in Bangladesh [9–11]. Ceftriaxone and azithromycin remained active and were used for treatment. There were no isolates resistant to ceftriaxone or ciprofloxacin (MIC ≥1.0 μg/mL) [12, 13]. The mortality in the confirmed enteric fever cases at 3.8 % was comparable with other studies of hospitalized patients [27–30]. We did not identify enteric fever caused by *S.* Paratyphi A in this group of patients, however this organism has been isolated from other febrile patients in Chittagong and at other sites in Bangladesh [8]. It may be that paratyphoid fever is less severe than typhoid in this setting and less likely to result in hospital admission, although observations across other parts of South Asia suggest that typhoid and paratyphoid may be of equal clinical severity [31].

Lower respiratory tract infections were the second most common clinical syndrome and had a corresponding mortality of 10.4 %. A further 15 % of patients presented with non-specific febrile illness and there were no deaths in this group. CNS infections accounted for nearly 12 % of admissions and were responsible for 41 % of deaths. The etiology of these cases was undetermined in most patients mainly because CSF was not available for further analysis. Other identified organisms causing bacteremia included *Staphylococcus aureus*, *Streptococcus pneumoniae*, *E.coli, Enterobacter cloacae* and *Klebsiella pneumonia* which are typical causes of bacteremia in Asia [32]. The presence of meticillin resistant *Staphylococcus aureus* (MRSA) and extended spectrum beta-lactamase (ESBL) producing Enterobacteriacae illustrates the importance of resistance surveillance against commonly used antimicrobials in this region [33]. In Bangladesh, antimicrobials can be easily purchased in the community and most patients use them prior to seeking medical attention. More than half of patients in this study consumed one or more antimicrobial prior to a blood draw for microbiological culture. Pre-treatment with antimicrobials will clearly reduce the yield of bacterial pathogens detected in blood and other clinical specimens. The three cases of *Burkholderia cepacia* bacteremia in children under the age of 5 years were unexpected. This organism may be associated with environmental contamination of blood cultures, but in each case here was associated with signs of sepsis.

The detection of two cases of *Rickettsia typhi* (murine typhus) and one case of *Orientia tsustugamushi* (scrub typhus) emphasizes the occurrence of these under-recognized pathogens in this area [34]. A case series in Mymensingh in the north of Bangladesh reported 40 *Rickettsia* infections, this study included 24 patients (60 %) positive for scrub typhus, by the Weil-Felix test [35]. A further case series described seven Bangladeshi nationals in Singapore with murine typhus [36]. In a recent prospective seroepidemiologic survey across six major teaching hospitals in Bangladesh, including CMCH, an IgM enzyme-linked immunosorbent assay was used to detect recent exposure to *Rickettsia typhi* and *Orientia tsustugamushi*. The results indicated that 805 of 1209 (66.6 %) subjects were seropositive for *Rickettsia typhi* and 287 of 1209 (23.7 %) were seropositive for *Orientia tsutsugamushi* [15]. Although the detection of IgM is less specific than IgG and cross-reactivity with antigens of other organisms may potentially lead to an over-estimate of the levels of infection, these studies suggest that these pathogens may be an important and under-reported cause of febrile illness in Bangladesh.

There were no confirmed cases of *Burkholderia pseudomallei* bacteremia despite previous reports of indigenous cases and in returning travelers [37, 38]. In a recent seroepidemiologic survey of six hospitals across Bangladesh, 359 of 1244 (28.9 %) of patients were seropositive for *B.pseudomallei* by indirect hemagglutination assay suggesting that many people may be exposed to the organism [16]. Also notable by its absence was leptospirosis despite serological evidence that it is a cause of febrile illnesses in this area [39, 40]. The absence of these infections in this study may be because the surveillance of febrile patients was not conducted throughout the the whole year covering all seasons. This is an important limitation of this study as is the small sample size and the limited range of diagnostic testing performed. The lack of CSF samples for further testing was a particular gap as patients diagnosed with a CNS infection were associated with the highest mortality. The results of this study should be therefore be regarded as preliminary and future studies should incorporate a wider panel of diagnostic methods for relevant pathogens and a minimum of a year-long recruitment period to encompass all potential seasonal variation.

## Conclusions

Enteric fever was a common cause of a non-malaria febrile illness in patients admitted to hospital in Chittagong. All isolated *S.* Typhi exhibited intermediate susceptibility to ciprofloxacin and many were MDR. Infection with *O. tsutsugamushi* and *R. typhi* were additionally confirmed in this setting. Lower respiratory tract and CNS infections were also common and CNS infections had a particularly high mortality rate with more than one third of patients dying during their hospital stay. The etiology of infections in this setting, in particular CNS infections, require further study.

## Abbreviations

ALT: Alanine transaminase
API: Analytical profile index
AST: Aspartate transaminase
ATCC: American type culture collection
CI: Confidence interval
CLSI: Clinical and Laboratory Standards Institute
CMCH: Chittagong Medical College Hospital
CNS: Central nervous system
CSF: Cerebrospinal fluid
DNA: Deoxyribose nucleic acid
EDTA: Ethylenediaminetetraacetic acid
ELISA: Enzyme linked immunosorbent assay
ESBL: Extended-spectrum beta-lactamase
IgM: Immunoglubulin M
IQR: Inter-quartile range
MDR: Multidrug resistant
MIC: Minimum inhibitory concentration
MRSA: Meticillin resistant *Staphylococcus aureus*
NS1: Dengue Non-structural protein 1
OUCRU: Oxford University Clinical Research Unit
PCR: Polymerase chain reaction
